# Nafion and Multiwall Carbon Nanotube Modified Ultrananocrystalline Diamond Microelectrodes for Detection of Dopamine and Serotonin

**DOI:** 10.3390/mi12050523

**Published:** 2021-05-06

**Authors:** An-Yi Chang, Shabnam Siddiqui, Prabhu U. Arumugam

**Affiliations:** 1 Institute for Micromanufacturing (IfM), Louisiana Tech University, Ruston, LA 71272, USA; aychang73@gmail.com; 2 Center for Biomedical Engineering and Rehabilitation Science (CBERS), Louisiana Tech University, Ruston, LA 71272, USA; 3 Department of Chemistry and Physics, Louisiana State University Shreveport, Shreveport, LA 71115, USA; Shabnam.Siddiqui@lsus.edu

**Keywords:** neurochemical, electrochemistry, microfluidic, carbon nanotube, diamond, microsensors, nafion

## Abstract

Neurochemicals play a critical role in the function of the human brain in healthy and diseased states. Here, we have investigated three types of microelectrodes, namely boron-doped ultrananocrystalline diamond (BDUNCD), nafion-modified BDUNCD, and nafion–multi-walled carbon nanotube (MWCNT)-modified BDUNCD microelectrodes for long-term neurochemical detection. A ~50 nm-thick nafion–200-nm-thick MWCNT-modified BDUNCD microelectrode provided an excellent combination of sensitivity and selectivity for the detection of dopamine (DA; 6.75 μA μM^−1^ cm^−2^) and serotonin (5-HT; 4.55 μA μM^−1^ cm^−2^) in the presence of excess amounts of ascorbic acid (AA), the most common interferent. Surface stability studies employing droplet-based microfluidics demonstrate rapid response time (<2 s) and low limits of detection (5.4 ± 0.40 nM). Furthermore, we observed distinguishable DA and 5-HT current peaks in a ternary mixture during long-term stability studies (up to 9 h) with nafion–MWCNT-modified BDUNCD microelectrodes. Reduced fouling on the modified BDUNCD microelectrode surface offers significant advantages for their use in long-term neurochemical detection as compared to those of prior-art microelectrodes.

## 1. Introduction

Understanding the role of neurochemicals in the functioning of the human brain in both healthy and diseased states is critical to the development of new and effective therapies for numerous neurodegenerative diseases [1]. Abnormal levels of neurochemicals such as dopamine (DA), serotonin (5-HT), glutamate, and GABA are the primary cause of epilepsy, Parkinson’s disease, traumatic brain injury, drug addiction, and many others [2,3,4]. DA is an important catecholamine in the mammalian central nervous system [3], because it is a central player in the brain “reward” system and plays a critical role in various bodily functions, i.e., motor control, motivation, and cognition, and in several debilitating neuropathologies [1,4]. Levels of 5-HT are linked to depression, addiction, and other functions ranging from appetite to sleep [5]. Electrochemical microsensors have been successfully employed to investigate the role of neurochemicals in real time [6]. Since DA and 5-HT are electrochemically active, they are readily and directly measured at physiologically relevant concentrations using electrochemical techniques such as cyclic voltammetry (CV), amperometry, differential pulse voltammetry (DPV), and fast-scan cyclic voltammetry with excellent spatial (micron range) and temporal (sub-second range) resolution in vitro and in vivo [7]. These methods routinely use carbon-fiber microelectrodes and glassy carbon electrodes with sub-micromolar sensitivity [8,9]. Among the emerging electrode materials for electrochemical microsensors, carbon nanomaterials such as carbon nanotubes [10,11,12,13], carbon nanofibers [14,15,16], micro- and nanocrystalline diamond [17,18,19] are frequently used to detect neurochemicals with either high sensitivity and/or high selectivity. Single-wall and multi-wall carbon nanotubes (SWCNTs and MWCNTs, respectively) have been widely used to detect neurochemicals by significantly increasing electroactive/adsorption sites for higher sensitivity and electrocatalytic/defect-rich sites for higher selectivity detection. In most cases, nanotubes have been used to modify existing electrodes such as carbon-fiber microelectrodes, graphite, glassy carbon electrode, carbon paste, and diamond-like carbon to increase adsorption sites, decrease oxidation overpotentials and improve sensitivity [4,9,10,11,12].

One of the grand challenges for the chemical neuroscience field is to develop a neurochemical microsensor that has a useful lifetime in the order of several hours or days, so that a more meaningful understanding of brain disorder mechanisms can be gained [20]. The useful lifetime of carbon-nanomaterial-enabled microsensors is generally extended due to their high resistance from chemical etching and little surface fouling due to by-products such as melanin and dimers generated from neurochemical oxidation [21,22]. The carbon-fiber microelectrode, which is the current gold-standard electrode material, is not suitable for chronic neurochemical recording due to their susceptibility towards surface fouling and degradation [22,23]. On the contrary, previous studies have shown that conductive boron-doped polycrystalline diamond has excellent electrochemical properties—superior chemical inertness and dimensional stability, a wide electrochemical potential window, extremely low background currents, exceptional biocompatibility for brain chemical sensing [1,13,14], and most importantly, greater surface fouling resistance than other forms of nanocarbon electrode materials [24].

In this study, we have microfabricated and fully characterized a nafion-coated hybrid MWCNT film-modified boron-doped ultrananocrystalline diamond (BDUNCD) microelectrode for long-term DA and 5-HT detection in the presence of ascorbic acid (AA). Nafion was chosen to block anionic molecules such as AA, although with an increase in the response time of analyte measurements [25]. A 2-µm-thick BDUNCD thin film was chosen as the bare microelectrode material because of its unique nanoscaled structure—ultra-small equiaxed grains (2–5 nm in diameter) and inherently ultra-smooth surface (R_a_: ~5–8 nm root mean square (rms)) [12]. Several groups including ours have used microlithographic techniques to produce well-defined, reproducible microelectrode geometries on BDUNCD films and wires for in vitro and in vivo neurochemical measurements [15,16,17,18,19,26]. MWCNTs were chosen as a modifying layer for the BDUNCD electrode surface because of its ballistic electronic properties, high surface area, excellent interfacial adsorption properties, and enhanced electrocatalytic activity. Several techniques have been employed previously to modify surfaces with carbon nanotubes, namely, chemical vapor deposition, drop casting, and electrophoretic deposition (EPD) [21,22]. Chemical vapor deposition processes are quite expensive, involving cumbersome microfabrication processes, costly cleanroom equipment, and high temperature growth processes that severely limit electrode and electrode substrate material choices [22,23,24,25,26,27]. Drop casting neither controls the thickness nor achieves a highly selective, uniform coating thickness on microelectrode surfaces [22]. However, EPD is well suited to deposit charged particles such as nanotubes with highly controllable coating thicknesses and the precise integration of the coating onto the microelectrode surface [24]. In this work, MWCNT films were selectively coated on 250-µm-diameter BDUNCD microelectrodes using EPD.

Furthermore, we have integrated a microfluidic platform to study the surface modified BDUNCD microelectrodes and changes in their sensor performance metrics for up to 9 h. Previous studies have integrated microfluidics with chemical and biological sensors because of their ability to perform multiplexed real-time measurements rapidly with reduced sample volumes, enhanced analyte transport, increased analyte sensitivity, automation, and lower costs [28,29,30,31]. Specifically, it provides the capability to inject controllable amounts of different neurochemicals onto the microsensor surface to evaluate its performance more accurately. In this work, we have employed a droplet-based microfluidics platform to evaluate the sensor metrics (sensitivity, response time, clearance rate, selectivity, and limit of detection (LOD)) in detail with controllable neurochemical flow rates and volumes and sub-second-to-second changes in the levels of the neurochemicals at the electrode surface that is expected in the brain [32].

## 2. Materials and Methods

### 2.1. Microfabrication of the BDUNCD Microelectrode Array

**Figure 1:**
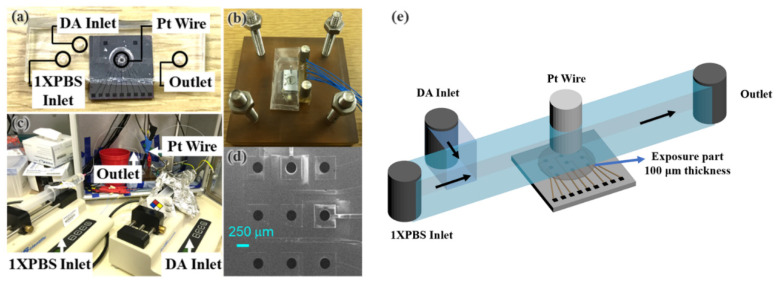
(**a**) Optical picture of an integrated boron-doped ultrananocrystalline diamond (BDUNCD) chip—microfluidic platform. (**b**) Optical picture of an assembled system with electrical connections. (**c**) Experimental setup showing connections to the micro syringe pumps and the potentiostat. (**d**) SEM image of a 3 × 3 BDUNCD microarray chip. (**e**) Schematic showing the integration of microfluidics with the BDUNCD chip.

The substrates employed for these microelectrodes were four-inch silicon wafers with a 1-μm-thick thermal silicon dioxide (Wafer World Inc., Palm Beach, FL, USA) surface coating. A 2-μm-thick BDUNCD film was then deposited with a hot filament chemical vapor deposition process from Advanced Diamond Technologies, Inc. (Romeoville, IL, USA). The BDUNCD film resistivity was ~0.08 Ω·cm as measured by a 4-point probe from a witness wafer (Pro4, Lucas Labs, Gilroy, CA, USA). The average roughness of the BDUNCD film was <10 nm rms based on AFM measurements (Digital Instruments, Santa Barbara, CA, USA). Optical microlithography was used to pattern 21 chips per wafer. Each chip was micro-patterned into nine individually electrically addressable 250-μm-diameter disk microelectrodes (geometrical area: ~0.05 mm^2^) in a 3 × 3 microelectrode array format, shown in Figure 1a,d (details described elsewhere) [12,33].

### 2.2. Preparation of Nafion and MWCNT Coatings Using EPD

Both MWCNT and nafion coatings (layers) were deposited by a two-electrode method. BDUNCD was the working electrode, and a platinum microwire (for MWCNT) and an Ag/AgCl electrode (for nafion) were the counter and the reference electrode, respectively. A 1 mg/mL MWCNT suspension in deionized (DI) water (PD15L-5-20, outside diameter (OD): 15 ± 5 nm, length: 1–5 μm, 5% –COOH functionalized; Nanolab, Inc., Waltham, MA, USA) was used for the coating. Before EPD, a 5 µM MgCl_2_·6H_2_O salt solution was added to the MWCNT suspension and sonicated for 30 min. This imparted a positive charge to the MWCNTs [34,35]. A ~30 µL MWCNT suspension was placed between the BDUNCD chip and the Pt counter electrode. A stepwise voltage scan (−6 V) was applied using a Gamry reference 600 Potentiostat (Gamry Instruments, Warminster, PA, USA) to the BDUNCD microelectrode for 10 min, and it was then cured at 70 °C for 10 min (details described elsewhere) [36]. A ~200-nm-thick MWCNT coating (via SEM imaging) was thereby achieved. For nafion coating, a 5 wt % solution (Sigma Aldrich, St. Louis, MO, USA) was used. A ~20 µL nafion solution was placed between the chip and the Ag/AgCl counter/reference. A stepwise voltage scan (+0.5 V) was applied to the microelectrode for 2 min followed by a rinse in deionized water and curing at 70 °C for 10 min. A ~50-nm-thick nafion coating (via SEM imaging) was thereby achieved.

### 2.3. Microfabrication of Microfluidic Devices

A two-layer polydimethylsiloxane (PDMS; Sylgard 184 with a precursor to curing agent weight ratio of 10:1 was purchased from Dow Corning, Inc., Midland, MI, USA) microfluidic chip was constructed and integrated into the BDUNCD chip (Figure 1) with excellent sealing and no fluid leakage. Firstly, the BDUNCD chip was cleaned by acetone, isopropyl alcohol, and deionized water and dried in an oven at 50 °C. Secondly, a 100-µm-thick PDMS (1st layer) was formed by mixing PDMS and the curing agent (at a 10:1 volume ratio) and allowing it to cure at 60 °C for 2 h. The PDMS on top of the microelectrode area and the areas where the fluidic parts were connected were removed using a punch blade (Figure 1a). Another PDMS layer (2nd layer) with a 65-µm thickness was formed on a SU8-2025 epoxy-based photoresist mold that defined the microchannel. This layer was microfabricated by mixing the PDMS and the curing agent (at a 10:1 volume ratio) and allowing it to cure at 60 °C for 4 h. Then, the PDMS layer with the patterned microchannel was peeled from the SU8 mold, and the inlet, outlet, and counter/reference access holes were fabricated with a punch blade. Finally, the 2nd PDMS layer was bonded to the 1st PDMS layer-BDUNCD chip assembly by applying an oxygen plasma treatment at 40 W for 20 s (UVP Blak-Ray™ B-100AP) [37]. The complete integration of microfluidics to the BDUNCD microarray chip is shown in Figure 1e.

### 2.4. Electrochemical Measurements

All electrochemical experiments were carried out with an Autolab potentiostat (PGSTAT 302N, Metrohm USA, Riverview, FL, USA) configured in a two-electrode setup with a Pt microwire coil (Alfa Aesar, Haverhill, MA, USA) counter/reference electrode. The BDUNCD, nafion-BDUNCD, or nafion-MWCNT-BDUNCD microelectrode was configured as the working electrode. The microelectrode surfaces were exposed to the analyte (single component or ternary mixtures) or 1X PBS electrolyte solutions that were pumped into the microfluidic channel using two micro-syringes pumps (KDS100 Infusion Pump—780100, Kats Scientific, Denton, TX, USA) (Figure 1b,c). At any given time during an experiment, we applied only one type of sample solution (e.g., 1X PBS, 1X PBS with DA, 1X PBS with 5-HT, or 1X PBS with a mixture of DA and 5-HT solutions) across all the 9 microelectrodes in the array as a continuous flow using one of the two inlets. The microelectrodes were fashioned in a 3 × 3 array format with a diameter of 250 μm (Figure 1d). For each microarray chip, the electrical isolation of the pads was measured using a two-point probe multimeter. This ensured the integrity of the silicon dioxide passivation, which was essential for stable electrochemical performance. Prior to characterization, the BDUNCD microelectrode array was briefly sonicated in ethanol for 30 s and dried in nitrogen. Differential pulse voltammograms were recorded with a 20-mV modulation amplitude and 5-mV step potential [38] for selectivity measurements. All experiments were repeated at least 3 times with three different microelectrodes (*n* = 3) for more than one chip. The DPV voltammograms shown below are representative of the data. All solutions were freshly prepared on the same day when the experiments were conducted and purged with nitrogen gas for 5 min before use.

## 3. Results and Discussion

### 3.1. Characterization of Unmodified and Nafion-, and Nafion–MWCNT-Modified BDUNCD Microelectrodes

**Figure 2:**
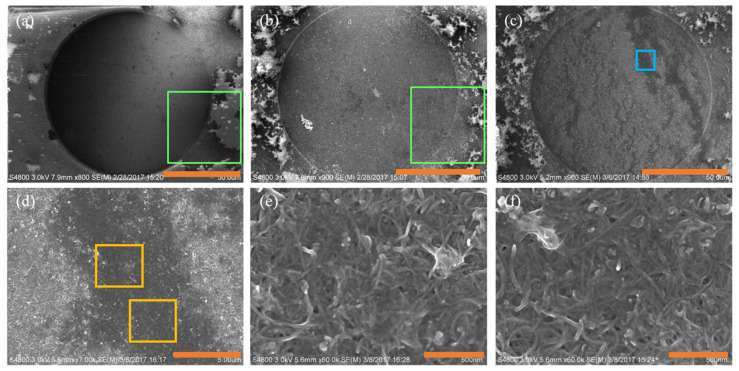
The SEM images of nafion- and nafion–multi-walled carbon nanotube (MWCNT)-coated BDUNCD microelectrodes: (**a**) unmodified BDUNCD microelectrode; (**b**) MWCNT-modified BDUNCD deposition at −6 V for 10 min; (**c**) nafion–MWCNT-modified BDUNCD microelectrode at +1.5 V for 2 min; (**d**) nafion–MWCNT-modified BDUNCD electrode at a high magnification image of the region shown in the blue square in (c); (**e**,**f**) the two yellow squares shown in (d). The scale bars for (**a**–**f**) are 50, 50, 50, 5, 0.5, and 0.5 µm, respectively.

**Figure 3:**
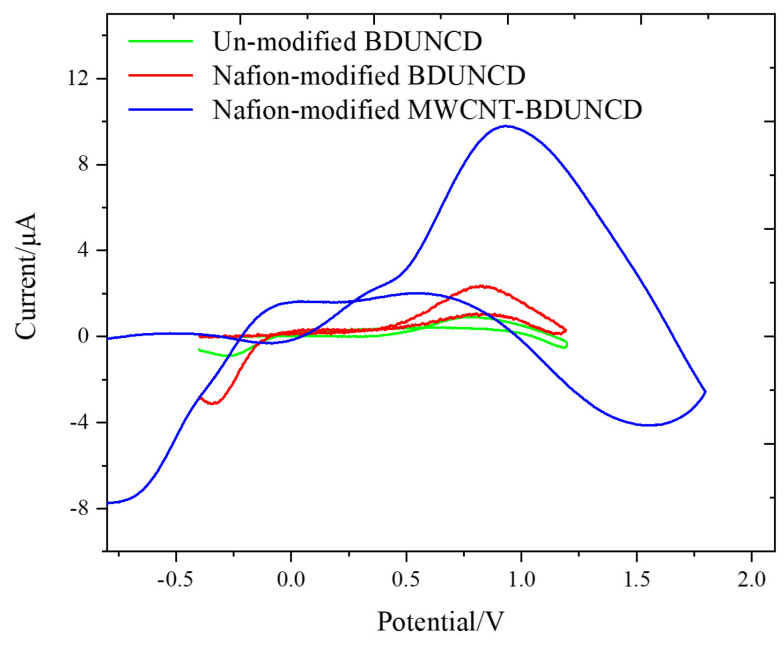
Fast-scan cyclic voltammograms of surface-modified BDUNCD microelectrodes. The green color represents the unmodified BDUNCD microelectrode, the red color represents the nafion-modified BDUNCD microelectrode (red), and the blue color represents the nafion–MWCNT-modified BDUNCD microelectrode. Voltammograms were taken in 100 µM dopamine (DA) and 1X PBS buffer at 400 V/s and undertook background subtraction. The flow rate was 0.2 mL/min.

Figure 2a,b displays the SEM images of an unmodified and MWCNT-modified BDUNCD microelectrode surfaces with MWCNTs covering almost the entire surface of the microelectrode. After nafion coating (Figure 2c), a heterogenous surface was observed as expected. During the nafion coating process, we observed a ~10% loss of non-specifically attached MWCNTs due to the application of a positive potential towards the BDUNCD working microelectrode. Nevertheless, a careful investigation of the nafion–MWCNT–BDUNCD surface at higher magnifications (Figure 2d–f) revealed a sufficient, uniform coverage of the two coatings, which is an important criterion for its application in long-term reliable neurochemical monitoring. The fast-scan cyclic voltammetry (FSCV) voltammograms are displayed in Figure 3 for the three types of BDUNCD microelectrodes in a 100 µM DA solution at a flow rate of 0.2 mL/min. Table 1 summarizes the important measured FSCV parameters of the microelectrodes. The peak oxidation current (I_pa_) that is commonly used as the detection signal was ~10-fold higher for the nafion–MWCNT--modified BDUNCD microelectrode than that of the unmodified BDUNCD microelectrode. The nafion coating on the unmodified BDUNCD surface demonstrated a ~4-fold higher current than that of the unmodified microelectrode. The oxidation peak potential (E_pa_) marginally increased from 0.78 ± 0.01 V for unmodified BDUNCD surfaces up to 0.94 ± 0.01 V for nafion–MCNCT-modified BDUNCD surfaces. The large increase in the current signals (and a higher sensitivity) is due to a significant increase in the electroactive area and sites achieved via MWCNT coating [36,39] and the anionic nature of nafion that is expected to adsorb the positively charged DA [18,40]. The results suggested the importance of coating both nafion and MWCNT for improving the overall sensitivity of diamond microelectrodes. The next question to investigate is whether the two-coating modification strategy can provide adequate sensitivity and selectivity long-term. For this, we employed a microfluidic setup to thoroughly characterize the three BDUCND microelectrode types using droplet microfluidics that mimics neurochemical discharges in the extracellular space of the brain [41].

### 3.2. Optimization of Droplet Parameters

We employed microfluidics to pass droplets containing a known analyte concentration over the microelectrode surface using a 1X PBS buffer as the carrying (background) medium. For droplet experiments, we still used only one type of solution but introduced them as droplets using both inlets. In this case, the analyte solution was pumped from one inlet, and the background 1X PBS was continuously pumped from the other inlet (as shown in Figure 1e). For example, during the 9-h study, we employed 1X PBS for the background continuous fluid flow and introduced the ternary mixture of DA, 5-HT, and AA as droplets at a predetermined frequency (1 droplet per min or 1 droplet every 2 min) and with a droplet volume of 0.02 mL. We allowed the droplets to flow over the microarray during the 9 h experiments. Periodically (every 3 h), we stopped the background flow and applied only the analyte solution to flow over the microarray and detected the analytes via DPV. The waiting time between droplets is 2 min, so that the analyte current signal can decrease to background levels. The change in the background currents was monitored for any fouling due to DA by-products such as melanin that was expected to form a thin passivation coating on the electrode surface with time [18]. In addition to the droplet flow rate, its volume is important to optimize and control, as it affects the analyte diffusion within the microchannel and thus, the amplitude and the stability of the current signal. The following optimal conditions that provided a stable signal and flow conditions (details published in 27) were chosen for this work: 0.1 mL/min (1X PBS) for the background flow rate, 0.02 mL for the droplet volume with 100 μM DA concentration, and +0.35 V for the applied potential (vs. a Pt microwire).

### 3.3. Evaluation of the DA Sensitivity, Response Time, and Clearance Rate Using Droplets

**Figure 4:**
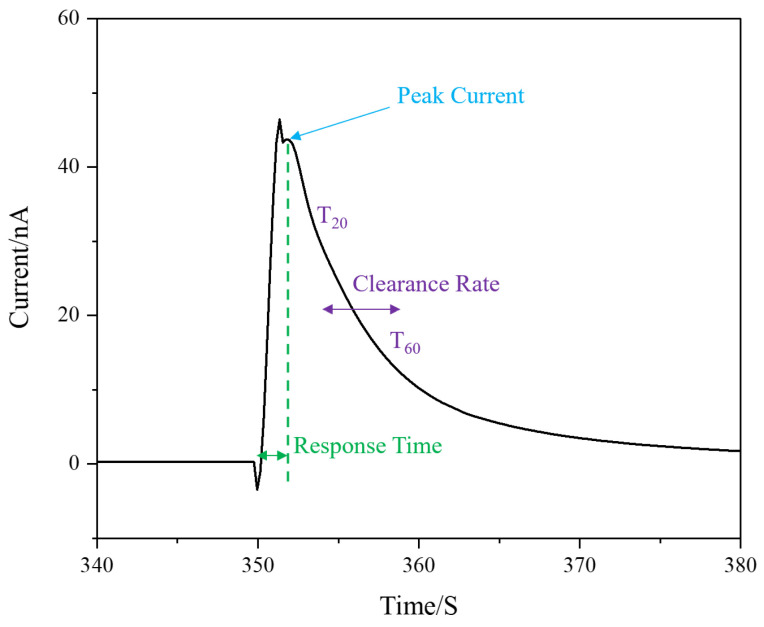
A sample DA signal arising from a single droplet flow across the microelectrode. The method shown was employed for calculating the sensitivity, response time, and clearance rate.

**Figure 5:**
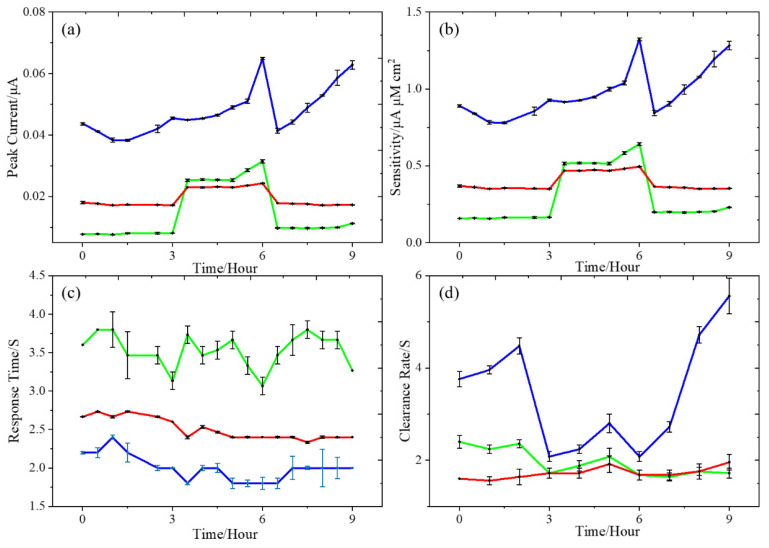
Long-term evaluation of three BDUNCD microelectrode types in terms of (**a**) peak currents, (**b**) sensitivity, (**c**) response time, and (**d**) clearance rates. Experimental conditions: 1 droplet per min with a background flow rate of 0.1 mL/min in 1X PBS) and 0.02 mL (100 μM, DA) droplet volume. The applied potential was +0.35 V vs. Pt microwire; every 30 min we took five signals and calculated the error bar in the 9-h experiment. The green color represents the unmodified BDUNCD microelectrode, the red color represents the nafion-modified BDUNCD microelectrode, and the blue color represents nafion–MWCNT-modified BDUNCD microelectrode.

The long-term changes in the key sensor metrics (peak current, sensitivity, response time, and clearance rate) as defined in Figure 4 is shown in Figure 5 for the three microelectrode types. During the experiments, five measurements were obtained every 30 min for up to 9 h. The microchannel width was 1800 μm, and the droplets’ volume was 0.02 mL (100 μM DA) with a background flow rate of 0.1 mL/min (1X PBS). Each droplet was introduced every 2 min into the microchannel and as present over top of the microelectrode for ~5 s. The DA’s oxidation peak currents were determined from the second peak, which did not vary by the mass transfer effects within the microchannel [18]. The first peak varied due to the mass transfer effect, which resulted in an unstable peak current signal. Other possible causes for the variation in the first peak’s oxidation potential variation are the flow conditions and changes in the input solution volume [18]. Therefore, for improved precision in the analysis, we considered only the second peak, which was stable irrespective of the changes in the flow conditions and volume. Sensitivity was defined as the peak current divided by the DA concentration and the electrode area (μA μM^−1^ cm^−2^). The time delay between the start of the rising peak and the second peak was used to calculate the response time. The time between 20 (T_20_) and 60% (T_60_) of the falling peak was used to calculate the clearance rate, i.e., the time for the DA signal to decrease to background levels.

#### 3.3.1. Sensitivity

The DA signal was stable during the first 3 h of the experiment for the unmodified BDUNCD microelectrode. However, the peak current signal and sensitivity increased about 3.4 times (0.16 ± 0.01 to 0.55 ± 0.05 μA μM^−1^ cm^−2^) in the next 3 h, which could be due to the activation and regeneration of BDUNCD grains and grain boundaries that was observed during our previous work (Figure 5a) [42]. In that work, we noticed an increase in BDUNCD grain capacitance or conductivity contributing to an increase in DA current. Beyond 6 h, we observed a sharp decrease in the currents and sensitivity due to the BDUNCD surface fouling as was identified during previous work Figure 5b [14]. The poly-dopamine was deposited onto the BDUNCD electrode surface with time, which caused the background current to shift to more negative values [14,18]. As expected, it gradually blocked the current signals via the formation of melanin [14]. However, the nafion-modified BDUNCD microelectrode exhibited a stable signal with a higher DA sensitivity (0.40 ± 0.06 μA μM^−1^ cm^−2^) when compared to the un-modified BDUNCD microelectrode (0.30 ± 0.18 μA μM^−1^ cm^−2^) with a constant background current for the entire 9 h recording. This could be due to a reduction in the rate of surface fouling, thus increasing the electrode’s useful lifetime. The negatively charged nafion layer rejected the negatively charged polydopamine, which otherwise fouled the microelectrode surface [18]. In addition, it reduced the chemical intermediates of o-dopaminoquinone (o-DQ), Leucodopaminochrome (LDC), and Dopaminochrome (DC) remaining on the surface that would otherwise form melanin and contributes to electrode fouling [14].

The nafion–MWCNT-modified BDUNCD microelectrode demonstrated the highest average currents and sensitivities, a 3-fold increase (0.97 ± 0.15 μA μM^−1^ cm^−2^) compared to the unmodified electrodes, and the sensitivity continued to increase during the entire 9 h recording (Table 2). This is due to a significant increase in the electroactive area as expected from the MWCNT layer [36,39], and the negatively charged nafion increased the adsorption of the positively charged DA. In addition, the nafion layer assisted in a reduction of the surface fouling. However, the signal varied continuously during the recording, and this could be due to the non-uniform, porous nature of the MWCNT layer with its inherent surface roughness and heterogeneity, which might affect the diffuse layer within the modified layers. 

#### 3.3.2. Response Time

Response time is defined as the time for the sensor to increase in current from the background current level to that of the second oxidation peak current [43]. The nafion–MWCNT-modified BDUNCD microelectrode is shown in Figure 5c, which had the fastest response time (2.0 ± 0.16 s) with good stability when compared to the unmodified BDUNCD microelectrode (3.5 ± 0.21 s) and the nafion-modified BDUNCD microelectrode (2.5 ± 0.15 s). Clearly, MWCNTs improved the response time, and this could be due to the ballistic electronic properties of MWCNTs [36,39]. A fast response time of less than a second to a few seconds is generally desirable for studying the in vivo neurotransmitter dynamics. These modified microelectrodes are excellent candidates for such demanding studies.

#### 3.3.3. Clearance Rate

The clearance rate is defined as the time between T_20_ and T_60_ (T_20_ is the signal reduced by 20% from the peak signal, and T_60_ is the signal reduced by 60% from the peak signal). From Figure 5d, the nafion-modified BDUNCD microelectrode had the fastest clearance rate (0.65 ± 0.01 s). However, the nafion–MWCNT-modified BDUNCD microelectrode had the slowest clearance rate (3.35 ± 1.81 s) compare to the unmodified BDUNCD microelectrode (1.7 ± 0.34 s). The reason for this could be due to the porous, high-surface-roughness heterogeneous nature of the modified layers. Currently, we are optimizing the two modified layers to achieve a faster clearance rate that is desirable for neurochemical monitoring.

### 3.4. Selectivity Measurements in a Ternary Mixture of DA, 5-HT, and AA

**Figure 6:**
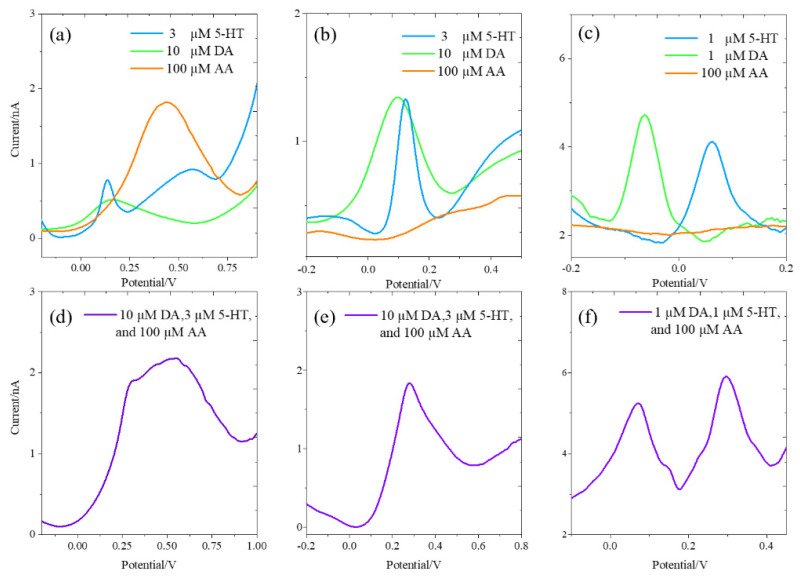
Differential pulse voltammetry (DPV) plots of the unmodified (**a**,**d**), nafion-modified (**b**,**e**), and nafion–MWCNT-modified (**c**,**f**) BDUNCD microelectrodes using individual analytes (**a**–**c**) and ternary mixture (**d**–**f**) in the solution. For nafion–MWCNT-modified BDUNCD microelectrodes, lower concentrations of DA and serotonin (5-HT) were used.

All the three microelectrode types detected DA and 5-HT individually (single component) using the DPV method (Figure 6a–c) although with different sensitivities. Only the unmodified BDUNCD microelectrode detected AA and as expected, not those coated with nafion. The oxidation potentials of DA, 5-HT, and AA on the unmodified BDUNCD electrode were 52.49 ± 2.62 mV, 82.7 ± 4.13 mV, and 47.5 ± 2.38 mV, respectively. However, AA exhibited a large, broad current peak that completely overlapped DA and 5-HT current signals (Figure 6a,d), and therefore the three current signals were undistinguishable (Figure 6d). Therefore, it is challenging for one to selectively detect DA and 5-HT in the presence of excess AA without electrode surface pre-treatment. In individual (single component) analyte solutions, the nafion-modified BDUNCD microelectrode demonstrated sharper and narrower DA and 5-HT current signals with reduced peak current values at 7.93 ± 0.40 mV and 77.66 ± 3.88 mV, respectively (Figure 6b). However, in a ternary (multiple component) mixture with AA present, selective detection of DA and 5-HT with distinguishable current peaks (Figure 6e) was not achieved. This is due to a wider DA oxidation potential, which varied from −164.03 ± 3.28 mV to 274.04 ± 5.48 mV and overlapped with the 5-HT signal. As expected, AA was rejected on the nafion-coated electrode, which therefore provided the necessary selectivity required for in vivo measurements. In contrast, the nafion–MWCNT-modified BDUNCD electrode exhibited sharper and narrower current peaks for both DA and 5-HT with the highest peak currents and sensitivities (Figure 6c) in individual analyte solutions. The oxidation potentials of DA and 5-HT were −83.47 ± 4.67 mV and 62.56 ± 3.13 mV, respectively. In ternary mixtures with AA present, the distinguishable peaks for DA and 5-HT were retained with excellent selectivity and with respective oxidation potentials at −12.97 ± 0.02 mV and 173.34 ± 2.52 mV [39] (Figure 6f). The peak oxidation current increased 5.5 times (0.11 ± 0.025 nA) for DA when compared to that of the unmodified BDUNCD microelectrode (0.02 ± 0.005 nA) and 3.3 times (0.46 ± 0.106 nA) for 5-HT when compared to the unmodified BDUNCD microelectrode (0.14 ± 0.033 nA) (Table 3). The DA and 5-HT sensitivities increased 165.5 times (3.31 ± 0.728 nA, 6.75 µA µM^−1^ cm^−2^) and 15.9 times (2.23 ± 0.468 nA, 4.55 μA μM^−1^ cm^−2^), respectively, when compared to the unmodified BDUNCD microelectrodes.

#### Long-Term DA and 5-HT Measurements

**Figure 7:**
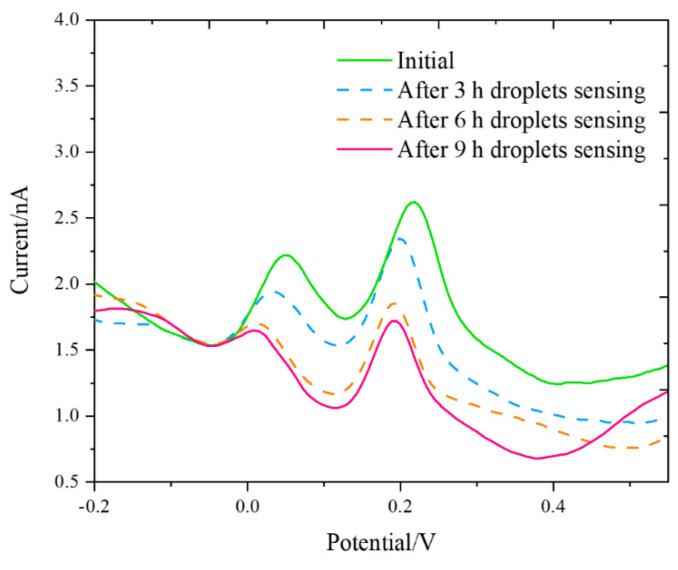
DPV plots of the nafion–MWCNT-modified BDUNCD microelectrode in a ternary mixture of 1 μM 5-HT, 100 μM AA, and 1 μM DA and at 0th, 3rd, 6th, and 9th h. The flow rate was 0.1 mL/min with a droplet volume of 0.02 mL, a background flow rate of 0.1 mL/min, and a potential of +0.35 V with a droplet frequency of 2 min.

A long-term study was conducted to evaluate the sensor metrics of nafion–MWCNT-modified BDUNCD microelectrodes (Figure 7). The DPV voltammograms for sampling times of 0, 3, 6, and 9 h in a ternary mixture of 1 μM DA, 1 μM 5-HT, and 100 μM AA were collected. Before the experiments, the microelectrodes were cleaned in a 1X PBS solution with a flow rate of 0.1 mL/min for 20 min. The initial DA and 5-HT peak currents were 0.58 ± 0.03 nA and 1.03 ± 0.06 nA, respectively, and the initial DA and 5-HT sensitivities were 1.18 μA μM^−1^ cm^−2^ and 2.09 μA μM^−1^ cm^−2^, respectively. After 3 h of continuous use, the DA and 5-HT signals decreased by 28% and 0.06%, respectively, which could be due to some loss of surface –COOH functional groups of the MWCNT layer that would aid in DA adsorption. However, after 9 h, the DA and 5-HT signals decreased by 47% and 26%, respectively. The fouling was much less significant when compared to our earlier work [14] on BDUNCD surface stability, where we observed a 50% reduction in DA signals during the 2 h intentional surface fouling via the amperometry detection method. In this work, we have demonstrated that by suitable surface modifications, BDUNCD microelectrode lifetime can be significantly increased (~4-fold). Further optimization of the coatings is in progress to reduce the surface loss and the sensor metrics in the future. We, therefore, have demonstrated the importance of modifying BDUCND microelectrodes with nafion and MWCNT and their utility for long-term DA and 5-HT detection.

### 3.5. LoD and Limit of Quantification (LoQ) for DA Detection

The LoD is the lowest analyte concentration for which a signal is likely to be reliably distinguishable from the Limit of Blank. The lowest DA concentration detectable on the nafion–MWCNT-modified BDUNCD microelectrode was 1 nM DA with a peak current of 0.06 ± 0.01 nA, and a LoD of 5.4 ± 0.40 nM. The LoD was calculated using Standard Deviation/slope times 3 [44], which was three times the standard deviation divided by the slope. The LoQ is the lowest concentration at which the analyte cannot only be reliably detected but at which some predefined goals for bias and imprecision are met. By varying the DA concentration from 1 nM to 50 nM, we measured the LoQ to be 18.9 ± 1.78 nM.

## 4. Conclusions

We developed a droplet-based microfluidic platform to investigate three types of BDUNCD microelectrodes modified with nafion and MWCNTs for long-duration high-sensitivity neurochemical measurements in vitro. The nafion-modified BDUNCD microelectrode exhibited the highest sensitivity and selectivity towards DA and 5-HT in the presence of excessive AA. We observed the least amount of surface fouling with good signal stability and significant current enhancement with a nafion coating. Remarkable DA and 5-HT sensitivity and selectivity were achieved by incorporating an MWCNT layer. Our nafion–MWCNT-modified BDUNCD microelectrode recorded a 166-fold and 16-fold increase in DA and 5-HT sensitivity, respectively (6.75 µA µM^−1^ cm^−2^ and 4.55 μA μM^−1^ cm^−2^, respectively) when compared to the unmodified BDUNCD microelectrode (0.04 µA µM^−1^ cm^−2^). Furthermore, we observed distinguishable DA and 5-HT current peaks during the long-term stability studies (up to 9 h) with the nafion–MWCNT electrode surface modification. The ability to monitor neurochemicals with excellent long-term sensitivity and selectivity will allow this modified diamond microelectrode to become an important tool for exploring the effects of stimulation and drugs on neuronal networks and for expanding our understanding of neuronal changes in the brain for both healthy and diseased states.

## Figures and Tables

**Table 1:** Comparison of 100 µM DA peak oxidation and reduction currents and potentials of the cyclic voltammograms from the three types of BDUCND microelectrodes (unmodified, nafion-modified, and nafion–MWCNT-modified BDUCND) (*n* = 3).

CV Parameters	BDUNCD	Nafion-BDUNCD	Nafion-MWCNT-BDUNCD
I_pc_ (µA)	−0.89 ± 0.04	−3.15 ±0.16	−7.87 ± 0.47
I_pa_ (µA)	0.92 ± 0.05	2.37 ± 0.12	9.79 ± 0.49
I_pc_/I_pa_	0.97	1.33	0.8
E_pc_ (V)	−0.29 ± 0.01	−0.35 ± 0.01	−0.8 ± 0.01
E_pa_ (V)	0.78 ± 0.01	0.82 ± 0.02	0.94 ± 0.01

**Table 2:** Comparison of DA sensitivity, response time, and clearance rate during 9 h on the BDUNCD, nafion-modified BDUNCD, and nafion–MWCNT-modified BDUNCD electrodes (*n* = 3).

Electrodes	9 h Sensitivity (μA μM^−1^ cm^−2^)	Response Time (s)	Clearance Rate (s)
BDUNCD	0.30 ± 0.18	3.5 ± 0.21	1.7 ± 0.34
Nafion-BDUNCD	0.40 ± 0.06	2.5 ± 0.15	0.65 ± 0.01
Nafion-MWCNT-BDUNCD	0.97 ± 0.15	2 ± 0.16	3.35 ± 1.81

**Table 3:** Comparison of DPV peak currents from individual (single component) analytes (1 μM DA, 1 μM 5-HT, and 100 μM ascorbic acid (AA)).

Electrode	DA Current (nA) (1 μM)	5-HT Current (nA) (1 μM)	AA Current (nA) (100 μM)	DA Sensitivity Value μA μM^−1^ cm^−2^
BDUNCD	0.02 ± 0.005	0.14 ± 0.033	0.17 ± 0.038	0.04
Nafion-BDUNCD	0.11 ± 0.025	0.46 ± 0.106	N/A	0.22
Nafion-MWCNT-BDUNCD	3.31 ± 0.728	2.23 ± 0.468	N/A	6.75

## Data Availability

Not applicable.
